# Adaptive Melanin Response of the Soil Fungus *Aspergillus niger* to UV Radiation Stress at “Evolution Canyon”, Mount Carmel, Israel

**DOI:** 10.1371/journal.pone.0002993

**Published:** 2008-08-20

**Authors:** Natarajan Singaravelan, Isabella Grishkan, Alex Beharav, Kazumasa Wakamatsu, Shosuke Ito, Eviatar Nevo

**Affiliations:** 1 Institute of Evolution, University of Haifa, Mount Carmel, Israel; 2 Department of Chemistry, Fujita Health University School of Health Sciences, Toyoke Aichi, Japan; Pasteur Institute, France

## Abstract

**Background:**

Adaptation is an evolutionary process in which traits in a population are tailored by natural selection to better meet the challenges presented by the local environment. The major discussion relating to natural selection concerns the portraying of the cause and effect relationship between a presumably adaptive trait and selection agents generating it. Therefore, it is necessary to identify trait(s) that evolve in direct response to selection, enhancing the organism's fitness. “Evolution Canyon” (EC) in Israel mirrors a microcosmic evolutionary system across life and is ideal to study natural selection and local adaptation under sharply, microclimatically divergent environments. The south-facing, tropical, sunny and xeric “African” slope (AS) receives 200%–800% higher solar radiation than the north-facing, temperate, shady and mesic “European” slope (ES), 200 meters apart. Thus, solar ultraviolet radiation (UVR) is a major selection agent in EC influencing the organism-environment interaction. Melanin is a trait postulated to have evolved for UV-screening in microorganisms. Here we investigate the cause and effect relationship between differential UVR on the opposing slopes of EC and the conidial melanin concentration of the filamentous soil fungus *Aspergillus niger*. We test the working hypothesis that the AS strains exhibit higher melanin content than strains from the ES resulting in higher UV resistance.

**Methodology/Principal Findings:**

We measured conidial melanin concentration of 80 strains from the EC using a spectrophotometer. The results indicated that mean conidial melanin concentration of AS strains were threefold higher than ES strains and the former resisted UVA irradiation better than the latter. Comparisons of melanin in the conidia of *A. niger* strains from sunny and shady microniches on the predominantly sunny AS and predominantly shady ES indicated that shady conditions on the AS have no influence on the selection on melanin; in contrast, the sunny strains from the ES displayed higher melanin concentrations.

**Conclusions/Significance:**

We conclude that melanin in *A. niger* is an adaptive trait against UVR generated by natural selection.

## Introduction

An adaptation is a phenotypic or genotypic feature that is functionally designed by natural selection and improves Darwinian fitness (e.g., survival and/or reproductive advantage) relative to alternative features [1]. Evolution occurs as a consequence of organisms adapting to different environments over space and time [2]. Therefore, demonstrations of such environment-fit adaptations in organisms are crucial for elucidating evolution in action. Adaptation is a causal consequence of divergent selection associated with different habitats or ecological niches [3]. Thus, studying the traits under divergent selection and the ecological factors responsible for it (agents of selection) is an essential step towards the understanding of local adaptation.

The “Evolution Canyon” (EC) model [4], [5] is an optimal natural field-lab in which the harmony between organisms and their environments can be explored. The model has ecologically divergent environments at a microscale level, conferring divergent selective pressures on the surviving organisms. The orientation and spatial structure of the opposing slopes of “Evolution Canyon” inflicts differential insulation of solar radiation on each slope [6]. The slopes share the same geology, soil type, and macroclimate. But they differ sharply in microclimatic and biotic conditions because of the higher solar radiation on the south-facing “African” slope (AS) compared to the north-facing “European” slope (ES) [6]. The difference in solar radiation, in turn, is driving the two opposite slopes, separated by 200 meters on average, into ecologically contrasting environments; the sunny, tropical, warm, and xeric south-facing slope (SFS) typifies the African biota found on the “African” Slope (AS), which receives 200–800% higher solar radiation than the shady, temperate, humid north-facing (NFS) slope characterized by European biota, referred to as the “European” Slope (ES) [5], [6]. This differing environmental aspect in EC obviously selects for different adaptations in the organisms' survival, and solar ultraviolet radiation (UVR) is one of the major selection agents in EC influencing the organism-environment interaction. Thus, EC provides a suitable model system to study local adaptations.

Here we investigate the specific adaptations of the filamentous soil fungus *Aspergillus niger* to different UV conditions on the opposite slopes of “Evolution Canyon” I (Lower Nahal Oren, Mount Carmel, Israel). *A. niger* was chosen as a candidate species for the study because it is one of the most common soil fungi documented from all parts of the world [7], [8] and reported from all Israeli soils subjected to mycological investigations [9]. Since melanin is a trait considered evolutionary-derived in fungi for UV resistance [10], [11], we examined how melanin concentrations and the survival of the *A. niger* strains from opposite slopes vary under differential exposure to solar UVR. Our working hypothesis is that the strains from the sunny “African Slope” (AS) exhibit higher melanin content and therefore are better able to resist UVR than strains from the shady “European” Slope (ES).

## Results

### Melanin concentration

**Figure 1:**
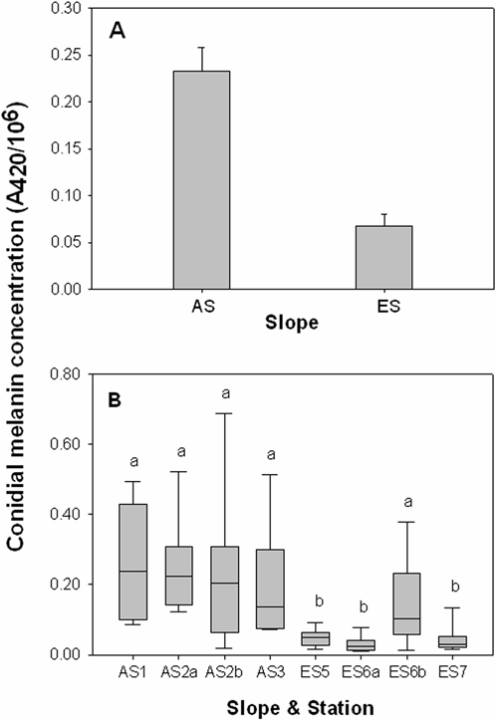
Melanin concentration in *A. niger* strains from “Evolution Canyon” I, Israel. (A) Mean (n = 40; stations and habitats pooled)±SE of AS and ES. (B) Box plot showing means (n = 10; typed by line within the box plot) and variations from six stations. AS2a and AS2b denote strains from sunny and shady microniches, respectively, whereas ES6a and ES6b indicate strains from shady and sunny micro- niches, respectively (the usage of “a” and “b” for ES6 is opposite to AS2 and is based on the order of predominant (a) and minor (b) habitats on the aforementioned stations). Means separation in columns by the Duncan's multiple range test, 5% level.

Mean melanin concentrations, measured as an absorbance at 420 nm in Soluene-350, was significantly different (t-test: t_(78)_ = 5.879, P<0.001) in the conidia of *Aspergillus niger* strains from the sunny AS (0.233±0.0254) than shady ES (0.0674±0.0123) by a factor of about 3 (Fig. 1A). There were also significant differences (One Way ANOVA with Duncan's multiple range test: F_(7, 72)_ = 5.871, P<0.001) found in melanin concentrations in strains from various stations in EC (Fig. 1B). Mean melanin concentrations of sunny (0.244±0.0407) and shady (0.229±0.0663) strains from AS2 (a predominantly sunny habitat) were not significantly different. However, a significant difference exists in melanin concentrations when strains from station ES5 (a predominantly shady habitat) were compared with sunny (0.146±0.0379) and shady (0.030±0.0068) microniches in ES5.

### UV-resistance

**Figure 2:**
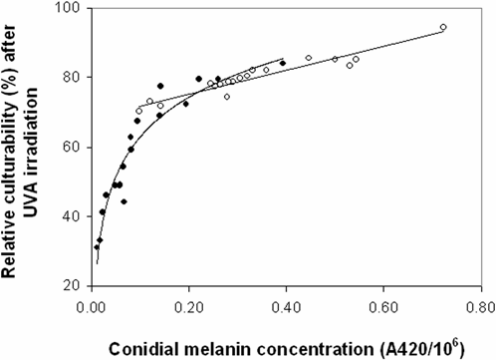
Relative culturability of *A. niger* strains after 4 h exposure to UVA irradiation plotted against conidial melanin concentration. Regression equations: linear for AS strains (open circles) y = 34.383x+68.414, R^2^ = 0.9166, P<0.001), and logarithmic curve for ES strains (solid circles) y = 16.334Ln(x)+100.67 R^2^ = 0.9187: p<0.001).

**Figure 3:**
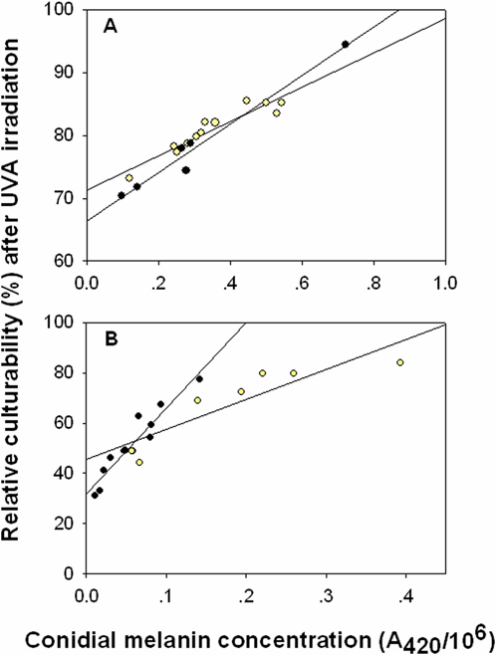
Relative culturability of *A. niger* strains after 4 h exposure to UVA irradiation plotted against conidial melanin concentration for AS (A) and ES strains (B) from sunny (yellow circles) and shady (solid black circles) microniches.

To investigate whether the adaptation of higher melanin concentration in the AS strains indeed leads to increased fitness, we studied the UV-resistance potential of selected strains (covering stations and habitats) from both slopes. The relative culturability after UVA irradiation of the AS strains (79.9%±1.36) was significantly higher than that of the ES strains (57.75%±3.71) (non-parametric Mann-Whitney rank sum test: T = 474, n_1_ = 18, n_2_ = 19, P<0.001) (Fig. 2). However, plots and regressions indicated that these differences were dependent on the positive relationship between relative conidial culturability and the concentration of melanin. The melanin content (A420/10^6^) values, within a range between 0.2 and 0.4, of strains from both slopes exhibit a fairly equal range of relative conidial culturability (75%–85%). The positive relationship between relative conidial culturability (after exposure to UVA) and concentration of melanin was indicated also by linear regression for both sunny and shady microniches. Regression equations for AS strains (Fig. 3A) were: y = 71.42+26.97x, R^2^ = 0.884, p<0.0001 in the sunny microniche; y = 66.31+38.75x, R^2^ = 0.973, p = 0 .0003 in the shady microniche. The slopes of the two regression lines were significantly different (t = 2.71, df = 14, P = 0.006). For ES strains (Fig. 3B): y = 45.46+119.57x, R^2^ = 0.801, p = 0.007 in the sunny microniche; y = 31.75+343.02x, R^2^ = 0.9089, p<0.0001 in the shady microniche. Slopes of the two regression lines were significantly different (t = 4.54, df = 15, P = 0.0004).

## Discussion

The present study is the first detailed experimental demonstration of specific adaptation in soil fungi indicating that melanin concentration in different strains does respond to differential solar UVR selection pressures on a microscale level. Highly melanic AS strains did resist UVA irradiation better. Shady (e.g., minor) conditions had no significant influence on melanin content in the AS strains (mainly exposed to sunny conditions), whereas sunny (e.g., minor) conditions inflicted clear selection pressure on ES strains (exposed mostly to shady conditions), in accumulating significantly higher melanin to cope with high solar UV radiation. Differences in melanin concentrations of strains from AS and ES were due to differential exposure to solar UV radiation rather than just due to soil temperature of the respective habitats. To summarize, it appears that AS strains and ES strains are adaptively radiated by higher and lower melanin content, respectively.

For a trait to be regarded as an adaptation it must be a derived trait that evolved in response to a specific selective agent [12], e.g., melanin and the selective agent - solar UV radiation seem to have a causal relationship. Higher melanin concentrations confer increased fitness to the AS strains, supporting the operational definition of adaptation that ‘it is a phenotypic variant that results in the highest fitness among a specified set of variants in a given environment [13].

UV radiation has been a ubiquitous feature/stressor of the exposed surface of earth since the origin of life itself. It is a damaging form of radiation that penetrates to the earth's surface and is therefore an important regulator of organism survival [14]. Sunlight consists of about 95% UVA radiation [15]. Melanin provides a fine example of physiological value of both inducible and constitutive defense against UV radiations [14], [16], [17]. The primary (evolutionary) function of melanin in microorganisms is to reduce the detrimental effects of UV radiation, e.g., DNA damage [14]. Albeit the correlation between concentration of melanin and UV tolerance is debated [14], our study clearly substantiates the causal relationships between melanin content and UV resistance. Despite the fact that high temperature augments melanin content in microorganisms [14], [16], this cannot be the case in our study because we have grown and maintained all strains at similar temperatures.

Fungal melanins are brown to black pigments formed by oxidative polymerization of phenolic compounds [10]. Though fungal melanins are not essential for normal growth, they enhance survival and competitive abilities of fungi in stressful environments [10], [11]. Melanin-containing fungi are well-known stress-tolerant microorganisms resistant to solar radiation, high temperature, water deficiency, oligotrophic conditions, and chemical and radioactive pollution [11], [18]. Dominance of dark-colored microfungi is characteristic for almost all mycologically studied desert soils [19]–[21]. Melanic fungi were also prevalent in the soils of sunny habitats of the “African” slopes in the northern Israeli “Evolution Canyons” [21].

Previous studies have shown deleterious effects of short-wavelength UV-B radiation (280–315 nm) on the survival and growth of terrestrial fungi [22]–[29]. Pigmentation of fungal mycelium and spores, especially pigments of melanin nature, appeared to play an essential role in UV protective mechanisms [11], [18], [30]. In the Israeli northern EC I and II, comparative UV-resistance was studied on the strains of the bacterium *Bacillus simplex* from the soil of opposite slopes (irradiation with a standard G15T8 lamp, UV-C, 254 nm, at a distance of 23 cm for 5 s). It was shown that the strains from the ES were significantly less UV-resistant than the strains from the AS [31]. Additionally, the sunny AS strains of *Bacillus simplex* exhibit higher melanin content than the shady ES strains from both ECI and ECII (Singaravelan et al. *in preparation*). Likewise, yeast strains of *Saccharomyces cerevisiae* from the AS were significantly more resistant to UVA radiation than ES strains (Gaby Lidzbarsky, Tamar Shkolnik, and Eviatar Nevo, in preparation). Thus, the “Evolution Canyon” microsite reveals multiple adaptations, including UV resistance in bacteria, fungi, and yeast, caused by the divergent microclimatic interslope stresses across life and represents an exceptional natural laboratory for unfolding adaptive radiation and incipient sympatric speciation [32].

## Materials and Methods

### Site description

**Figure 4:**
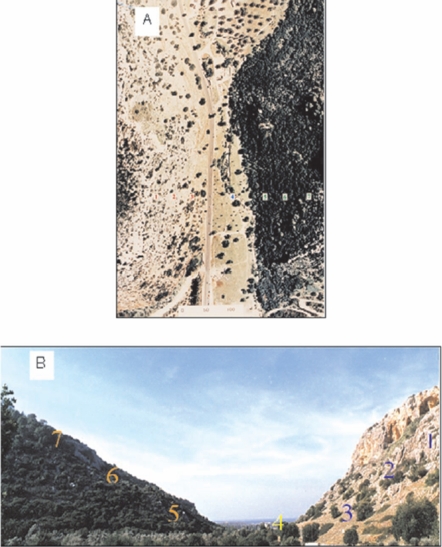
Evolution Canyon I (study system) Lower Nahal Oren, Mount Carmel, Israel, A. Aerial View, B. Cross section (Source: Science from Israel-LPPLtd).

“Evolution Canyon” I (EC I) (Fig. 4) is located at lower Nahal Oren (32°43′N; 34°58′E), a deeply incised valley running from Mount Carmel westward into the Mediterranean Sea. The climate of the region is characterized by mild rainy winters and dry summers, with mean annual rainfall ca. 600 mm and mean August and January temperatures of 28°C and 13°C, respectively [33]. The opposite slopes of the canyon have identical regional evolutionary history, geology, and soil type [4], [34], but they differ in topography (the south-facing “African” slope dips 35°; the north-facing “European” slope dips 25°). The slopes are separated by 100 m at the bottom and 400 m at the top, 200 meters apart, on average.

The climatic study in EC I [6] revealed that illuminance of the AS was significantly (2.3–8 times) higher than that of the ES; mean daily temperatures as well as daily temperature ranges were higher on the AS than on the ES; and, except under the high summer sun, relative humidity was 1–7% higher on the ES. The “African” south-facing slope (AS) is covered by xeric, Mediterranean, savannoid formation of the evergreen *Ceratonia siliqua-Pistacia lentiscus* and savanna grasses (*Hyparrhenia hirta*, *Andropogon distachyos*, and *Pennisetum ciliare*). In contrast, the “European” north-facing slope (ES) is covered by an east Mediterranean maquis-forest (brushwood forest) of evergreen live oak, *Quercus calliprinuos*, and deciduous *Pistacia palaestina* [35]. The dramatic interslope biotic divergence is illustrated by the fact that 62% of the higher plant species differ between the slopes and that the plant cover, making up the total cover of vegetation layers (measured by the Whittaker method) ranges from 35% (upper AS) to 150% (middle ES) [35].

### Sampling

Soil samples were collected in February 2006 from the upper soil layer (1–5 cm deep). The samples were taken at the upper, middle, and lower parts of the AS (stations 1, 2, and 3, respectively) and the ES (stations 7, 6, and 5, respectively). Within each station, the soil sample collection was random. We collected 10 samples from stations AS1, AS3 (sunny habitats); ES5, ES7 (shady habitats); and 20 samples from AS2 and ES6 (10 samples from sunny and 10 samples from shady microniches at each station). Altogether, 80 samples (40 from each slope) were collected.

### Isolation of *A. niger* strains

Strains were isolated using the soil dilution plate method [36]. Ten grams of each sample were initially diluted. One mL of sample suspension from the dilutions 1∶10 and 1∶1000 (soil: sterile water) was mixed with the malt extract agar medium (MEA) of the temperature near 40°C in Petri dishes of 90 mm diameter. Streptomycin was added to the medium (100 µg/ml) to suppress bacterial growth. The plates were incubated at 25°C (dilution 1∶1000) and 37°C (dilution 1∶10) in darkness for 7 days (two plates for each sample and for each temperature). After incubation, the colonies of *A. niger* were transferred to MEA for purification at 25°C and storage. Recent taxonomic studies using molecular methods described *A. niger* as a complex of morphologically indistinguishable species: *A. niger*, *A tubingensis*, *A. lacticoffeatus*, and *A. vadensis* [37]. Because we identified the studied strains based only on their morphological features, the set of strains could be heterogeneous and perhaps consist of molecularly different species. Details of the strains isolated are provided in supporting information file Appendix S1.

### Melanin assay

After 5 days of growth, the plates were flooded with Phosphate-Tween buffer (0.1 M potassium phosphate buffer [pH 7.0] containing 0.01% Tween 80) and gently rubbed with a sterile bent glass rod for conidial extraction. Spore suspensions were then removed and filtered through Schleicher & Schuell Rundfilter paper, retention size 12–25 µm. The washing and filtering procedure was repeated at least two times to achieve pure isolation of conidia. The filtrate was centrifuged for 10 min at 4000 rpm, and then the pellet was re-suspended in 1-ml phosphate buffer saline (PBS). The number of conidia per ml (for each strain) was quantified by counting under a microscope using a haemocytometer.

To measure total melanin concentrations, aqueous suspensions of the samples were sonicated with a sonicator (Handy Sonic UR-20P, TOMY SEIKO Co., Ltd., Tokyo, Japan) after adding 1 ml Milli-Q water. For a spectrophotometric characterization of samples, 900 µl Soluene-350 (Packard, Meriden, CT, USA) were added to aliquots of 100 µl and dissolved by heating in a boiling water bath for 45 min as described previously [38]. Absorbance of the resulting solutions was recorded on a Hitachi V-520 UV/VIS spectrophotometer. The absorbance at 420 nm per 10^6^ cells of sample reflects the total amount of melanin. The absorbance at 420 nm is based on results obtained by previous studies on *A. niger* and other congeners [39], [40].

### UV-resistance experiments

To examine UV-resistance, we chose 18 and 19 strains from different stations and with different melanin concentrations from the AS and the ES, respectively (for details see supporting information file Appendix S2). Fifty microliters of suspension with a concentration of 10^3^ conidia per ml (calculated by means of the haemocytometer) were spread over the surface of the plates with MEA. We prepared two sets of plates in triplicate, of which one set was assigned as the control and the other two as experimental replicates. Control plates were kept under room temperature in darkness. The opened experimental plates were irradiated with long wavelength ultraviolet light (UVA, 365 nm; 1200 µW/cm^2^ at 15″) from a high-intensity UV lamp (Spectroline model SB-100, Spectronics Corp., USA) for 4 hours, maintained at 25°C in a clean and sterilized milieu. The experimental plates were then placed in darkness along with the control plates. Both control and experimental plates were carefully monitored for the development of colonies. The relative culturability (%) after the UVA exposure was calculated for each strain as a mean number of colony forming units (CFU) on experimental replicates divided by the mean number of CFU on control replicates, multiplied by 100 [41]. The counts of CFU were made 48 h after UVA exposure.

### Statistical analysis

Numerical data are presented as means±SE. The t-test was employed to test the significance of differences in melanin concentrations in strains from the opposing slopes of EC. The differences in melanin concentrations between strains from various stations of EC were analyzed by the one-way analysis of variance, followed by the Duncan's multiple range test. Degrees of freedom are shown as subscripts both in Anova and t-test. The Mann-Whitney rank sum test was used to test the differences in relative culturability (%) after UVA irradiation between strains from the sunny AS and shady ES. The relationship between the concentration of melanin and the relative conidial culturability of the strains was determined by linear regression.

## Supporting Information

Appendix S1 (0.02 MB DOC)

Appendix S2 (0.05 MB DOC)
