# Supplementary material for: Adaptive Melanin Response of the Soil Fungus Aspergillus niger to UV Radiation Stress at “Evolution Canyon”, Mount Carmel, Israel
Source: PLoS One. 2008 Aug 20;3(8):e2993. doi: 10.1371/journal.pone.0002993 (PMC2500164; doi:10.1371/journal.pone.0002993)
Supplement: Appendix S1 — (0.02 MB DOC) [file pone.0002993.s001.doc]

Appendix S1 - Additional data on details of the *A. niger* strains

**“African” Slope (AS) – Contains predominantly sunny habitats**

Strains from Station 1 : AS1 _1-10_ (☼)

Strains from Station 2a-sunny (micro-niche) : AS2a _11-20_ (☼)

Strains from Station 2b-shady (micro-niche) : AS2b _21-30_ (•)

Strains from Station 3  : AS3 _31-40_ (☼)

**“European” Slope (ES) – Contains predominantly shady habitats**

Strains from Station 5 : ES5 _41-50_ (•)

Strains from Station 6a-shady (micro-niche) : ES6a _51-60_ (•)

Strains from Station 6b-sunny (micro-niche) : ES6b _61-70_ (☼)

Strains from Station 7 : ES7_71-80_ (•)

(☼) - Sunny

(•) - Shady

PS. Strain numbers are given in subscripts
