# Supplementary material for: Adaptive Melanin Response of the Soil Fungus Aspergillus niger to UV Radiation Stress at “Evolution Canyon”, Mount Carmel, Israel
Source: PLoS One. 2008 Aug 20;3(8):e2993. doi: 10.1371/journal.pone.0002993 (PMC2500164; doi:10.1371/journal.pone.0002993)
Supplement: Appendix S2 — (0.05 MB DOC) [file pone.0002993.s002.doc]

Appendix S2 – List of *A. niger* strains used in the UVA resistance experiments.

| “African” Slope (AS) | | “European” Slope (ES) | |
| --- | --- | --- | --- |
| Strain ID | Habitat/Micro niche | Strain ID | Habitat/Micro niche |
| AS1 _1_ | Sunny (☼) | ES5 _6_ | Shady (•) |
| AS1 _2_ | Sunny (☼) | ES5 _8_ | Shady (•) |
| AS1 _4_ | Sunny (☼) | ES5 _10_ | Shady (•) |
| AS1 _5_ | Sunny (☼) | ES6a _16_ | Shady (•) |
| AS2a _12_ | Sunny (☼) | ES6a _17_ | Shady (•) |
| AS2a _13_ | Sunny (☼) | ES6a _18_ | Shady (•) |
| AS2a _17_ | Sunny (☼) | ES6a _19_ | Shady (•) |
| AS2a _18_ | Sunny (☼) | ES6b _21_ | Sunny (☼) |
| AS2a _20_ | Sunny (☼) | ES6b _22_ | Sunny (☼) |
| AS2b _21_ | Shady (•) | ES6b _24_ | Sunny (☼) |
| AS2b _22_ | Shady (•) | ES6b _26_ | Sunny (☼) |
| AS2b _23_ | Shady (•) | ES6b _27_ | Sunny (☼) |
| AS2b _25_ | Shady (•) | ES6b _29_ | Sunny (☼) |
| AS2b _26_ | Shady (•) | ES6b _30_ | Sunny (☼) |
| AS2b _28_ | Shady (•) | ES7 _34_ | Shady (•) |
| AS3 _35_ | Sunny (☼) | ES7 _35_ | Shady (•) |
| AS3 _36_ | Sunny (☼) | ES7 _38_ | Shady (•) |
| AS3 _39_ | Sunny (☼) | ES7 _39_ | Shady (•) |
|  |  | ES7 _40_ | Shady (•) |
